# Eating behaviour of Indonesian adolescents: a systematic review of the literature

**DOI:** 10.1017/S1368980020002876

**Published:** 2021-06

**Authors:** Cut Novianti Rachmi, Hafizah Jusril, Iwan Ariawan, Ty Beal, Aang Sutrisna

**Affiliations:** 1 Reconstra Utama Integra, Jakarta, Indonesia; 2 Faculty of Public Health, Universitas Indonesia, Depok, West Java, Indonesia; 3 Global Alliance for Improved Nutrition (GAIN), Washington, DC, USA; 4 Department of Environmental Science and Policy, University of California, Davis, CA, USA; 5 Global Alliance for Improved Nutrition (GAIN), Menara Palma, 7th floor, Suite 705, Jl. HR Rasuna Said Blok X-2 Kav.6, Jakarta 12950, Indonesia

**Keywords:** Indonesia, Adolescents, Eating behaviour, Dietary intake, Food consumption

## Abstract

**Objectives:**

Global evidence has shown that behaviour acquired during adolescence often lasts into adulthood. Diet quality of and malnutrition in Indonesian adolescents is a neglected area of research. The current study reviews all studies related to eating behaviour in Indonesian adolescents to support evidence-based policy to improve diets.

**Design:**

We searched electronic databases (six international and one local), from January 2000 to April 2018. The search terms used were (1) prevalence (prevalence OR number* OR case*, incidence OR survey), (2) adolescents (adolescen* OR school-age OR young adult), (3) Indonesia (Indonesia*) and (4) eating pattern (eat* OR fruit OR vegetable OR food recall OR food OR frequenc* OR consumption OR dietary intake). Articles were assessed against a critical appraisal tool.

**Setting:**

Indonesia.

**Participants:**

10–19 years.

**Results:**

We discovered 15 studies related to eating behaviour, 5 of which were secondary analyses of nationally representative surveys and one was a nationwide survey. Of the nine studies, one study was conducted in multiple cities, and the rest were conducted in a single city or smaller area. There were seven main topics from the included studies: nutrient adequacy, fruit and vegetable consumption, water and beverage intake, Na intake, breakfast habit, snacking frequency and western fast food consumption.

**Conclusions:**

Adolescents consume inadequate amounts of protein, fruits and vegetables, and excessive amounts of Na and western fast food. Measures are needed to improve and motivate adolescents to adopt healthier eating patterns. Furthermore, there is a need to have one standard definition and measurement of eating behaviour in Indonesia.

Adolescent malnutrition is a growing global concern. Behaviour acquired during adolescence often lasts into adulthood^(1)^ and influences peers^(2)^. For some, health risks such as obesity and its psychological cost may also persist in adulthood^(3)^. Adolescents’ nutritional status plays an essential role in the health of their offspring, particularly for girls^(4,5)^. Thus, adolescence is a critical period to instill positive dietary and health behaviours. Moreover, particularly, urban adolescents are relatively more adaptive to new changes compared with other age groups^(6)^. Hence, examining adolescent dietary patterns may reflect changes occurring in the community^(2)^, especially for those nations going through the double burden of malnutrition.

The double burden of malnutrition – a condition in which both under- and over-nutrition occurs – is widespread in many countries, especially in low- and middle-income countries (LMIC)^(7,8)^. The fourth most populated nation globally, Indonesia, is still dealing with undernutrition while experiencing increasing overweight/obesity^(9–12)^. The Indonesia Basic Health Research (*Riskesdas*) survey reported that 25·7 % of adolescents aged 13–15 years were classified as stunted in 2018, a decrease from 35·1 % in 2013. Similarly, the proportion of thinness in adolescents 13–15 years reduced from 14·1 % in 2013 to 8·7 % in 2018. Overweight or obesity increased from 10 % in 2013 to 16 % in 2018. A similar pattern is occurring in older age groups. Among individuals 16–18 years, the nationwide survey reported a decrease in stunting (31·2 % in 2013 to 26·9 % in 2018) and thinness (19·4 % in 2013 to 8·1 in 2018). On the other end of the malnutrition spectrum, overweight or obesity increased from 7·3 % in 2013 to 13·5 in 2018^(13–15)^. A closer look is needed to support an evidence-based decision on this growing and transitioning nation. Previous pieces of evidence suggest that diets in Indonesia are becoming less healthy^(11,16)^. However, evidence on the adolescent group remains unexplored in Indonesia.

In Indonesia, the fact that adolescents will make up a growing proportion, demonstrating its significance, has led to more attention to this particular age group^(17)^. Nevertheless, there has been little implementation of programmes targeting adolescents; most programmes focus on adolescent girls, particularly on Fe and folic acid supplementation^(18)^. Anecdotal evidence suggests that the lack of attention over the last decade has left a paucity of nutrition-related data on adolescents in Indonesia. Using evidence from other countries to inform local measures is not enough because of the vast differences between countries^(2)^. The call for more evidence on adolescent health has been made since a decade ago, yet not enough progress has been made^(19)^. This literature review aims to identify and synthesise all published studies on the dietary patterns in Indonesian adolescents. Such a review is crucial to inform policies and programmes for all levels of government in Indonesia and to identify research priorities.

## Methods

### Inclusion and exclusion criteria

**Fig. 1:**
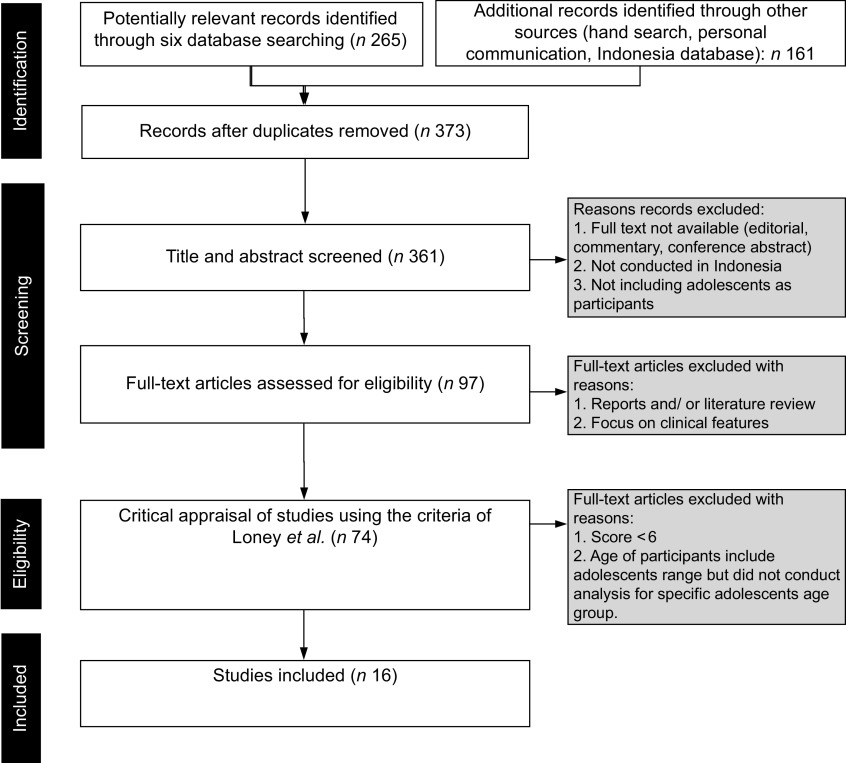
A flow diagram showing the flow of citations reviewed in the current study

The inclusion criteria were (a) studies that included people living in Indonesia aged 10–24 years^(20)^ as participants, (b) studies that investigated eating behaviour, (c) studies available in full text and (d) studies that were reported in English or in the Indonesian language published in journals (first and second authors speak Bahasa Indonesia and therefore are able to assess the quality and understand the results of these studies). We included studies of any design. The exclusion criteria were editorials or commentaries, studies only published as conference abstracts, those published only as reports or literature reviews and studies focusing on clinical features. Additional exclusion criteria are available in Fig. 1.

### Literature searching

We performed electronic searches in several databases: Medline via OvidSP, Global Health via OvidSP, Embase via OvidSP, Health Collection via Informit Online, Web of Science and Scopus from January 2000 to April 2018. The search used the following terms: (1) prevalence (prevalence OR number* OR case*, incidence OR survey), (2) adolescents (adolescen* OR school-age OR young adult), (3) Indonesia (Indonesia*) and (4) eating pattern (eat* OR fruit OR vegetable OR food recall OR food OR frequenc* OR consumption OR dietary intake). The results were then combined from 1 to 4 with AND. The search in Indonesian databases used similar/translated terms. Articles written in the Indonesian language were searched through Indonesia National Institute of Health Research and Development portal which consists of twenty-two local health journals. We also traced included articles forwards on Google Scholar and performed a hand search of included article references to check for additional articles that may not have been identified during the systematic search. The search was run twice for clarity and double-checked for quality.

### Document screening

After collecting all articles identified from the search, we removed duplicates. Articles were screened against inclusion criteria; relevant full texts were retrieved for further assessment to ensure quality. Titles and abstracts were screened by two researchers. After final exclusion of articles according to our inclusion and exclusion criteria, relevant papers were ready for quality appraisal.

### Critical appraisal

All full-text articles were screened against a tool by Loney *et al*.^(21)^. This tool appraised manuscripts based on three key indicators: validity of the study methods (six points), interpretation of results (one point) and applicability of results (one point). A complete table of the tool is available in online Supplementary Table 1. We only included studies that had a score more than or equal to six points. Two authors conducted the critical appraisal, and the third author resolved any disagreements. To ensure the quality of our systematic review process, we refer to the PRISMA checklist to guide us in writing this review (prisma-statement.org).

### Synthesis of result and data extraction

We present results in narrative synthesis, which allowed for an in-depth discussion of the qualitative heterogeneity between included studies. Results were further classified into studies conducted at the national-level, multi-city and single city or smaller area. We also classified results based on the topics discussed in the studies.

## Results

### Characteristics of studies

Throughout this literature review, we used 10–24 years as the age group for adolescents as proposed by Sawyer *et al*.^(20)^ on the search and screening process. However, most of the studies only included information on adolescents 10–19 years of age, fitting the WHO criteria. The complete process of screening is outlined in Fig. 1. Of the 15 included studies, one scored 8, four scored 7 and ten scored 6. The result of our appraisal process is available in Table 1.

**Table 1:** Critical appraisal process using Loney *et al*.^(21)^

Title of article (authors)	Random sample or whole population	Unbiased sampling frame	Adequate sample size (>300)	Measures were the standard	Outcomes measured in an unbiased fashion	Adequate response rate (70 %), refusers described	CI, subgroup analysis	Study subjects described	Score
National									
Fruits and vegetables consumption and associated factors among in-school adolescents in five Southeast Asian countries (Peltzer and Pengpid^(24)^)	Y	Y	Y	Y	Y	Y	Y	Y	8
Intake of water and beverages of children and adolescents in 13 countries (Guelinckx *et al*.^(30)^)	Y	Y	Y	Y	Y	Y	N	Y	7
Consumption of fruits and vegetables in the context of balanced nutrition among Indonesian: individual food consumption survey analysis 2014 (Hermina and Prihatini^(25)^)	Y	Y	Y	Y	Y	Y	N	Y	7
Food contribution in sodium intake of children at young age in Indonesia (Prihatini *et al*.^(31)^)	Y	Y	Y	Y	Y	N/A	N	Y	6
Sodium intake among Indonesian population: analysis of Individual Food Consumption Survey 2014 (Prihatini *et al*.^(32)^)	Y	Y	Y	Y	Y	N/A	Y	Y	7
Indonesian food consumption against balanced nutrition value (Safitri *et al*.^(23)^)	Y	Y	Y	Y	Y	N/A	Y	Y	7
Multi-cities									
Knowledge, attitudes and adolescent behaviour on nutrition-conscious families (Kadarzi): with special attention on weight monitoring and consuming diverse foods (Sudirman and Jahari^(22)^)	Y	Y	Y	Y	Y	N/A	N	Y	6
Single city									
Determinants of anemia in high school students in Jakarta (Ernawati and Saidin^(27)^)	Y	Y	Y	Y	Y	N/A	Y	Y	6
Factors associated with fruits and vegetables consumption among adolescent in 4 state high schools in West Jakarta (Bahria and Triyanti^(28)^)	Y	Y	N	Y	Y	Y	N	Y	6
Risk of western fast food consumption and skipping breakfast against obesity: a study at SMAN 1 Cirebon (Banowati *et al*.^(33)^)	Y	N/A	N	Y	Y	Y	Y	Y	6
Relationship of breakfast habits and hydration status with concentration of thinking in adolescents (Lentini and Margawati^(34)^)	Y	Y	N	Y	Y	N/A	Y	Y	6
Breakfast habits, nutrition status, and quality of youth life of Bosowa Junior High School Bina Insan Bogor (Niswah *et al*.^(35)^)	Y	Y	N	Y	Y	Y	N	Y	6
Risk factors for breakfast and snack on overweight events in high school adolescents (Agusanty *et al*.^(36)^)	Y	Y	N	Y	Y	N/A	Y	Y	6
Adolescents can prevent overweight with increasing self-efficacy and vegetable–fruit consumption (Widianto *et al*.^(29)^)	Y	Y	N	Y	Y	N/A	Y	Y	6
A social cognitive theory-based programme for eating patterns and sedentary activity among overweight adolescents in Makassar, South Sulawesi: a cluster randomised controlled trial (Hidayanty *et al*.^(37)^)	Y	Y	N	Y	Y	N/A	Y	Y	6

Y = Yes; N = No; N/A = Information not available in the paper.

Six studies were conducted at the national level, only one was conducted in multiple cities in Indonesia and eight were conducted in one city or smaller in scale. Ten of these studies were conducted in schools and six were conducted in non-school settings. The earliest study was published in 2008 and the latest in 2017.

### Level of studies

From six national studies available, two of them were conducted in several countries and four were conducted nationwide (secondary data analysis of nationally representative surveys). Two studies were non-school based and four school based. A more complete overview of these studies is available in Table 2.

**Table 2:** Studies included in the literature review based on level of studies

No.	Title of article (authors)	Study site	Time survey conducted	Survey setting	Subjects	Topic	Dietary pattern measures	Study design
	National							
1	Fruits and vegetables consumption and associated factors among in-school adolescents in five Southeast Asian countries (Peltzer and Pengpid^(24)^)	Global School-Based Health Survey in 5 South East Asian countries: India, Indonesia, Myanmar, Sri Lanka and Thailand	2008	School based	13–15- year students	Fruits and vegetables consumption	Questionnaire consumption frequency in the past 30 d, compared against recommended at least two daily servings of fruits and three daily servings of vegetables	Cross-sectional
2	Intake of water and beverages of children and adolescents in 13 countries (Guelinckx *et al*.^(30)^)	13 countries: Latin America (Mexico, Brazil, Argentina, Uruguay), Europe (Spain, France, Belgium, UK, Poland, Turkey) and Asia (Iran, China, Indonesia)	Between 2008 and 2014, year conducted in Indonesia is not detailed	School based	Junior and senior high school students	Salty, sweet food/sugary drinks	7-d records on fluid intake, followed by face-to-face interview	Cross-sectional
3	Consumption of fruits and vegetables in the context of balanced nutrition among Indonesian: Individual Food Consumption Survey 2014 analysis (Hermina and Prihatini^(25)^)	Nationwide (Studi Konsumsi Makanan Individu 2014)	2014	Non-school based	5–12 years, 13–18 years, 19–55 years	Fruits and vegetable consumption	24 h food recall, results compared against Indonesia RDA (*Pedoman Gizi Seimbang*)	Cross-sectional
4	Food contribution in sodium intake of children at young age (6–18 years) in Indonesia (Prihatini *et al*.^(31)^)	Nationwide (Studi Konsumsi Makanan Individu 2014)	2014	Non-school based	6–18 years	Na and salt consumption	24 h food recall	Cross-sectional
5	Sodium intake among Indonesian population: analysis of Individual Food Consumption Survey 2014 (Prihatini *et al*.^(32)^)	Nationwide (Studi Konsumsi Makanan Individu 2014)	2014	Non-school based	5–12 years 13–18 years	Na and salt consumption	24 h food recall, results compared against Indonesia recommended 2 grams Na intake per day	Cross-sectional
6	Indonesian food consumption against balanced nutrition value (Safitri *et al.* ^(23)^)	Nationwide (Studi Konsumsi Makanan Individu 2014)	2014	Non-school based	5–12 years 13–18 years	Food diversity, proportion and adequacy of consumption	24 h food recall, results compared against Indonesia RDA (*Pedoman Gizi Seimbang*)	Cross-sectional
	Multi-cities							
7	Knowledge, attitudes and adolescent behaviour on nutrition-conscious families (Kadarzi): with special attention on weight monitoring and consuming diverse foods (Sudirman and Jahari^(22)^)	West Java, West Sumatera, East Kalimantan, West Nusa Tenggara, South Sulawesi	N/A	Non-school based	10–19 years	Nutrition education and nutrients consumption	Questionnaire, consume protein, fruit and vegetables ≥ 5 times/week	Cross-sectional
	Single city or smaller area							
8	Determinants of anemia in high school students in Jakarta (Ernawati and Saidin^(27)^)	Jakarta	2005	School based	15–19 years senior high school students	Fruits, vegetables, meats consumption	FFQ, consumption frequency in the past week	Cross-sectional
9	Factors associated with fruits and vegetables consumption among adolescent in 4 public high schools in West Jakarta (Bahria and Triyanti^(28)^)	West Jakarta	2009	School based	Senior high school students	Fruits and vegetables consumption	Questionnaire, results compared against recommended twice a day consumption in a week	Cross-sectional
10	Risk of western fast food consumption and skipping breakfast against obesity: a study at SMAN 1 Cirebon (Banowati *et al*.^(33)^)	Cirebon	2010	School based	Senior high school students	Fast-food consumption	FFQ, consumption frequency in a month	Case–control (obese–non obese)
11	Relationship of breakfast habits and hydration status with concentration of thinking in adolescents (Lentini and Margawati^(34)^)	Surakarta	2014	School based	Senior high school students	Breakfast	Questionnaire, breakfast routinely	Cross-sectional
12	Breakfast habits, nutrition status, and quality of youth life of Bosowa Junior High School Bina Insan Bogor (Niswah *et al*.^(35)^)	Bogor	2014	School based	Junior high school students	Breakfast	24 h food recall, breakfast routinely	Cross-sectional
13	Risk factors for breakfast and snack on overweight events in high school adolescents (Agusanty *et al*.^(36)^)	Pontianak	2013	School based	Senior high school students	Breakfast and snacking	FFQ, breakfast routinely, carbs on snack compared against recommended < 105·6 g for boys and 89·4 g for girls	Case–control (overweight–normal weight)
14	Adolescents can prevent overweight with increasing self-efficacy and vegetable–fruit consumption (Widianto *et al*.^(29)^)	Jagakarsa	N/A	School based	Grade 7 and 8 junior high school students	Fruits and vegetables consumption	Questionnaire, results compared against four servings consumption per day	Cross-sectional
15	A social cognitive theory-based programme for eating patterns and sedentary activity among overweight adolescents in Makassar, South Sulawesi: a cluster randomised controlled trial (Hidayanty *et al*.^(37)^)	Makassar	N/A	School based	11–15 years junior high school students	Snacking	FFQ, snacking frequency	Cross-sectional

Table 2 shows that there was only one study available in the multi-cities level, a cross-sectional study conducted in five cities throughout Indonesia. The current study was conducted in non-school-going adolescents aged 10–19 years.

We discovered eight studies conducted in a single city or even smaller area, all of which surveyed school-going adolescents. Of these eight studies, five surveyed high school students and three junior high school students.

### Topics covered in the included studies

**Fig. 2:**
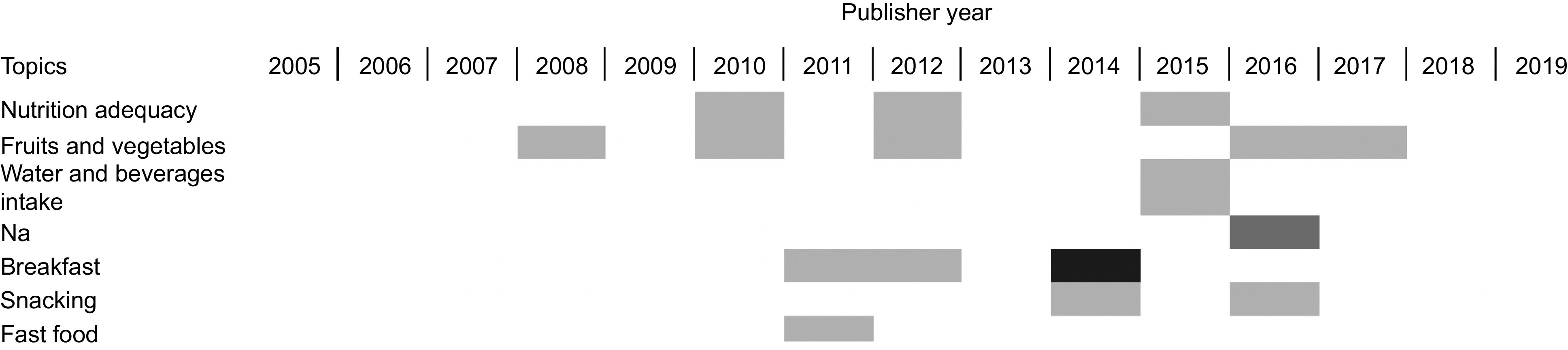
Trends of topics in adolescents’ eating behaviour explored on published papers. 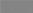, one study; 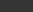, two studies; 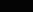, four studies

Of the 15 included articles on eating behaviour/patterns among adolescents in Indonesia, we also divide them based on the topics covered in each study. The earliest study was published on 2008 and more published studies found by 2014 (Fig. 2). There were three studies on breakfast, three on food consumption (including fruit, vegetable, and meat consumption), one on western fast food consumption and one on snacking (Table 2). Some of these studies covered more than one sub-topic; thus, we have more than fifteen studies listed in Table 3.

**Table 3:** List of studies around eating behaviour/patterns among adolescents conducted in Indonesian adolescents based on topics

Level of study	Topic	Authors	Results	
Nutrients adequacy				
National	Indonesian food consumption against balanced nutrition value^(46)^	Safitri *et al.*	Consume ≥ 4 food groups 6–12 years: 81·1 % 13–19 years: 83·9 % Adequate consumption (80–120 % AKG): 6–12 years: 38·1 % 13–19 years: 27·1 %	Good proportion of carbohydrate, protein, fat consumption (60:15:25): 6–12 years: 23·5 % 13–19 years: 24·9 %
Multi-cities	Knowledge, attitudes and adolescent behaviour on nutrition-conscious families (Kadarzi): with special attention on weight monitoring and consuming diverse foods^(22)^	Sudiman and Jahari	10–19 years; consume protein, fruit and vegetables ≥5 times/week: 18·8 %	10–19 years; consume multivitamin containing vitamin A: 9·6 %
Fruits and vegetables				
National	Fruits and vegetables consumption and associated factors among in-school adolescents in five Southeast Asian countries^(24)^	Peltzer and Pengpid	13–15 years Fruits and vegetables <5 servings/d: 75·2 %	13–15 years Most of the times or always went hungry: 5·8 %
National	Consumption of fruits and vegetables in the context of balanced nutrition among Indonesian: Individual Food Consumption Survey Analysis 2014^(25)^	Hermina and Prihatini	Inadequate consumption of fruit and vegetables; 13–18 years: 98·4 %	
Single city	Determinants of anemia in high school students in Jakarta^(27)^	Ernawati and Saidin	15–19 years, vegetables consumption in the past week Never: 6·3 % Sometimes: 54·4 %	15–19 years, fruits consumption in the past week Never: 7·3 % Sometimes: 69·5 %
Single city	Factors associated with fruits and vegetables consumption among adolescent in 4 state high schools in West Jakarta^(28)^	Bahria and Triyanti	Consume fruits < 2 times/d: 92·1 %	Consume vegetables < 2 times/d: 77·1 %
Single city	Adolescents can prevent overweight with increasing self-efficacy and vegetable–fruit consumption^(29)^	Widianto *et al*.	Fruits and vegetables consumption < 4 servings/d: 76·3 %	
Water and beverages intake				
National	Intake of water and beverages of children and adolescents in 13 countries^(30)^	Guelinckx *et al*.	10–17 years, mean ml/d (sd) Water: Male: 1621 (872) Female: 1589 (812)	10–17 years, mean ml/d (sd) Milk: Male: 73 (140) Female: 74 (166)
			10–17 years, mean ml/d (sd) Hot beverages: Male: 129 (224) Female: 101 (168)	10–17 years, mean ml/d (sd) Juices: Male: 13 (41) Female: 27 (87)
			10–17 years, mean ml/d (sd) Regular soft beverages: Male: 200 (334) Female: 203 (389)	
Na				
National	Food contribution in sodium intake of children at young age (6–18 years) in Indonesia^(31)^	Prihatini *et al*.	6–18 years, > 2000 mg/d: Male: 55·3 % Female: 54·2 % Total: 55·3 %	6–18 years, Na source: Salt: 43·9 % Food: 56·1 %
National	Sodium intake among Indonesian population: analysis of Individual Food Consumption Survey 2014^(32)^	Prihatini *et al*.	5–12 years, ≥ 2000 mg/d: 55·2 % 13–18 years, ≥ 2000 mg/d: 55·7 %	5–12 years, Na source: Salt: 43·3 % Food: 56·7 % 13–18 years, Na source: Salt: 44·1 % Food: 55·9 %
Breakfast				
Multi-cities	Knowledge, attitudes and adolescent behaviour on nutrition-conscious families (Kadarzi): with special attention on weight monitoring and consuming diverse foods^(22)^	Sudiman and Jahari	10–19 years, breakfast: 88·6 %	
Single city	Risk of western fast food consumption and skipping breakfast against obesity: a study at SMAN 1 Cirebon^(33)^	Banowati *et al*.	Breakfast Obese: 78·9 %Non-obese: 94·7 %	
Single city	Relationship of breakfast habits and hydration status with concentration of thinking in adolescents^(34)^	Lentini and Margawati	15–19 years, breakfast: 52·5 %	
Single city	Breakfast habits, nutrition status, and quality of youth life of Bosowa Junior High School Bina Insan Bogor^(35)^	Niswah *et al*.	13–15 years, breakfast: 83·3 %	
Single city	Risk factors for breakfast and snack on overweight events in high school adolescents^(36)^	Agusanty *et al*.	Breakfast Overweight: 11 % Non-overweight: 28 %	Routine high-carb breakfast Overweight: 36·4 % Non-overweight: 7·1 % (OR 7·5; *P* = 0·04)
Snacking				
Single city	Risk factors for breakfast and snack on overweight events in high school adolescents^(36)^	Agusanty *et al*.	Snacking > 2 times/d Overweight: 62 % Non-overweight: 46 % (OR = 1·9; *P* = 0·023) High-carb snacking Overweight: 31 % Non-overweight: 9 % (OR = 4·5; *P* = 0·0001)	High energy snacking Overweight: 44 % Non-overweight: 27 % (OR = 2·1; *P* = 0·012)
Single city	A social cognitive theory-based programme for eating patterns and sedentary activity among overweight adolescents in Makassar, South Sulawesi: a cluster randomised controlled trial^(37)^	Hidayanty *et al*.	11–15 y, median of snacking habit frequency score 0·50 (0·27 ± 0·99) (3–6 times/week)	
Western fast food				
Single city	Risk of western fast food consumption and skipping breakfast against obesity: a study at SMAN 1 Cirebon^(33)^	Banowati *et al*.	Frequency of western fast food consumption/month Obese: 27·9 ± 20·8 Non-obese 20·8 ± 16·9	Energy intake of western fast food consumption (kcal/d†) Obese: 263 ± 184 Non-obese: 140 ± 126
			Energy contribution from western fast food consumption ( % total kcal/d†) Obese: 8·4 ± 5·2 Non-obese: 6·2 ± 5·1	

† To covert kcal to kJ multiply it by 4·184.

#### Nutrient adequacy

There were two studies addressing nutrient adequacy. One study was conducted in Integrated Health Posts (Posyandu) in six cities in Indonesia, involving more than 900 unmarried adolescents aged 10–19 years^(22)^. The authors combined the consumption of animal-source protein and fruits and vegetables in one category. The other category was consumption of a multivitamin that included vitamin A. The results showed that only 18·8 % of adolescents consumed animal-source protein, fruits and vegetables more than 5 d in a week, with the highest prevalence in South Sulawesi (31·7 %) and the lowest in East Nusa Tenggara (3·8 %). The national prevalence of adolescents consuming a multivitamin containing vitamin A was 9·6 %. Among the subnational level, the highest prevalence was found in East Nusa Tenggara province (13·4 %), and the lowest was in West Nusa Tenggara province (6·3 %)^(22)^.

Another study analysed the nationwide Basic Health Survey and found notable inadequate consumption among adolescents (38·1 % among 6–12 year olds and 27·1 % among 13–19 year olds)^(23)^. Most adolescents already consumed ≥ 4 food groups (81·1 % among 6–12 year olds and 83·9 % among 13–19 year olds). However, only a few, 23·5 and 24·9 % of those aged 6–12 and 13–19 years, respectively, consumed an appropriate proportion of carbohydrate, protein and fat (60:15:25).

#### Fruit and vegetable consumption

There were five studies that addressed fruit and vegetable consumption. A secondary data analysis using the Global School-based Health Surveys 2008 dataset was conducted by Peltzer and Pengpid in five Southeast Asian countries, including Indonesia^(24)^. The study in Indonesia involved more than 2800 participants aged 13–15 years. The current study revealed that 75·2 % of adolescents in Indonesia consumed fruits and vegetables less than five servings per day in the past 30 d. They also analysed food insecurity, defined as going hungry due to lack of food at home in the past 30 d, and categorised the response to never, rarely, sometimes, most of the times and always. Results showed that 5·8 % of adolescents responded ‘most of the times’ or ‘always went hungry’ in the past 30 d^(24)^.

The next study was a secondary data analysis of Individual Food Consumption Survey (SKMI) in Indonesia^(25)^. The current study involved more than 15 000 adolescents aged 13–18 years. They used the recommended intake according to the Balanced Nutrition Guidelines (Pedoman Gizi Seimbang)^(26)^ which state the minimally adequate fruit and vegetable consumption is 400 g/d (250 g vegetables and 150 g fruits). Indonesia’s Balanced Nutrition Guidelines refer to the WHO guidelines for fruit and vegetable intake. The consumption under this cut-off was considered inadequate. In this age group, 94·7 and 28·9 % stated they consumed vegetables and fruits, respectively, each day. However, the amount of vegetables and fruits consumed was 62·1 and 106·6 g, respectively. An estimated 98·4 % of adolescents aged 13–18 years was considered to have inadequate intake of fruits and vegetables^(25)^.

Another included study performed secondary data analysis on the Survey of School-Children Nutritional Status in Indonesia^(27)^. From ten available cities in the survey, the authors focused in Jakarta city. The analysis included 491 students aged 15–19 years and asked about the frequency of fruit and vegetable consumption in the past week (never, sometimes or every day). The frequencies of vegetable consumption in the past week were 6·3, 54·4 and 39·3 %, respectively. The frequencies of fruit consumption in the past week were 7·3, 69·5 and 23·2 %, respectively. Among those who consumed fruits and vegetables, most consumed them sometimes rather than never or every day^(27)^.

Another study involved 214 students from four high schools in West Jakarta^(28)^. The current study measured consumption of fruits and vegetables in the past week and categorised consumption as adequate if the students consumed two servings per day (fourteen servings/week) of each item. Only 7·9 and 22·9 % of students consumed adequate fruits and vegetables in the last week, respectively. Factors that affected these prevalences were the amount of pocket money (associated with fruit consumption) and whether students liked vegetables (associated with vegetable consumption)^(28)^. Widianto *et al*. conducted a study of 156 students in grades seven and eight from junior high schools in Jagakarsa^(29)^. They considered adequate consumption of fruits and vegetables as consumption of four or more servings per day. The results showed that 76·3 % of these students had inadequate consumption of fruits and vegetables^(29)^.

#### Water and beverages intake

There is only one study of water and beverage intake in adolescents, and it was conducted in thirteen countries, including Indonesia^(30)^. The current study included 595 adolescents aged 10–17 years. The results showed that in both male and female adolescents, the most consumed beverage is water (regular and tap), followed by regular soft beverages (sugared and artificially sweetened, carbonated and non-carbonated soft drinks, energy drinks, sports drinks, other sugared or artificially sweetened soft drinks), hot beverages (coffee, tea and other hot beverages), milk (and milk derivatives) and juices^(30)^.

#### Sodium intake and source

A secondary data analysis of Individual Food Consumption Survey (SKMI) in Indonesia was conducted by Prihatini *et al*.^(31)^. The results revealed that in adolescents aged 13–18 years, 44·3 % of Na intake was consumed from salt and 55·7 % from other food ingredients. In all age groups (6–18 years), 55·3 % of participants consumed more than 2 g/d of Na. Another secondary analysis of Individual Food Consumption Survey (SKMI) reported similar results: 55·2 and 55·7 % of the population aged 5–12 and 13–18 years, respectively, consumed ≥ 2 g of Na per d. Of all populations (0 to > 55 years), individuals aged 13–18 and 19–55 years consumed the most Na, that is, 2·8 g/d^(32)^.

#### Breakfast habit

We found five studies addressing breakfast habits of adolescents. The first study, conducted by Sudiman and Jahari in Integrated Health Posts (Posyandu) in six cities in Indonesia, revealed that as many as 88·6 % of adolescents aged 10–19 years have breakfast regularly. The highest prevalence was found in West Nusa Tenggara (92·3 %) followed by East Kalimantan (90·5 %), East Nusa Tenggara (89·2 %), West Java (88·3 %), South Sulawesi (87·7 %) and West Sumatera (84·6 %)^(22)^. Banowati *et al*. conducted a case–control study involving seventy-six senior high school students, thirty-eight of whom were classified as obese and categorised to the case group^(33)^. They found that 78·9 % of obese students and a higher percentage of those who were not obese (94·7 %) had breakfast in the morning. There was also a significant difference (*P* = 0·019) between the frequency that obese students had breakfast compared with their non-obese counterparts (4·5 times/week *v*. 5·8 times/week, respectively)^(33)^.

Another study on breakfast habits was performed by Lentini and Margawati, where they randomly sampled eighty female high school students aged 15–19 years in Surakarta. They found that 47·5 % of these students were not used to having breakfast in the morning^(34)^. The next study was also conducted in school, targeting junior high school students aged 13–15 years in Bogor^(35)^. The author found that 16·7 % of students were not used to having breakfast in the morning. Although the number of students who were used to having breakfast was much higher (83·3 %), there was no significant difference in nutritional status between the two groups^(35)^. The last study that observed breakfast habits was also a case–control study, involving 200 students (100 overweight students as case group)^(36)^. A higher prevalence of students who routinely had breakfast was found among those who were not overweight (28 %) compared with their overweight counterparts (11 %).

#### Snacking frequencies

A study by Agusanty *et al*. highlighted a significant difference (*P* = 0·023) in the frequency of snacking more than two times per day between those who were overweight (62 %) and those who were not overweight (46 %). Overweight adolescents were also twice more likely to consume higher energy content snack (OR 2·1; *P* = 0·012) and fourth times more likely to consume high-carb snacks (OR 4·5; *P* = 0·0001). Instant noodles, vegetable deep fired fitters (*bakwan*) and candies were the top three most frequently consumed snacks in both groups. Overweight adolescents also frequently snack on fried rice and chocolate, meanwhile non-overweight group opted for banana fritters and tofu fritters^(36)^. Another study observed the snacking habits of 172 students from eight schools in Makassar and found that students snacked 3–6 times/week^(37)^.

#### Western fast food consumption

The study by Banowati *et al*.^(33)^ highlighted the difference between the frequency of consuming western fast food between obese (27·9 times/month) and non-obese (20·8 times/month) students, although the difference was not significant (*P* = 0·180). We found a significant difference (*P* = 0·001) in the energy intake from western fast food consumption: 1100·4 ± 769·9 kJ/d (263 ± 184 kcal/d) for the obese group and 585·8 ± 527·2 kJ/d (140 ± 126 kcal/d) for their non-obese counterpart. Samples of western fast food include pizza, fried chicken, french fries and a doughnut.

## Discussion

Our literature review revealed fifteen studies about eating behaviour among Indonesian adolescents. Of these, six were conducted nationwide, one in multiple cities and eight in a single city or smaller area. Each study used different definitions and/or measurements of eating pattern variables; thus, statistical comparisons between studies were challenging. We categorised the topics discussed in the included studies into seven main topics: nutrient adequacy, fruit and vegetable consumption, water and beverage intake, Na intake, breakfast habits, snacking frequencies and western fast food consumption.

Evidence suggests overall eating habits of Indonesian adolescents need improvement. About 18 % of sampled adolescents in six cities consumed animal-source protein, fruits and vegetables less than 5 d/week^(22)^. This finding, however, needs to be interpreted carefully, as only animal-source protein was included, while the Indonesian national guidelines suggest consuming 2–4 portions of plant-source or animal-source protein daily^(26)^. All studies revealed inadequate fruit and vegetable consumption, in either serving size and/or frequencies^(24,25,27–29)^. A secondary analysis of a nationally representative survey recorded 98·4 % adolescents aged 13–18 years consumed inadequate fruits and vegetables (< 400 g/d)^(25)^. These findings suggest attention is needed on diet quality in addition to energy adequacy among adolescents.

A systematic review of diets among adolescent girls in LMIC reported only half of adolescents consumed vegetables and fewer (37 %) consumed them daily, despite a high variation between regions. The highest proportion of daily vegetable consumption was recorded among Middle East and North African adolescent girls (82 %) followed by South Asia (72 %), Africa (5 %) and Latin America and the Caribbean (1 %). About 73 % of adolescent girls living in East Asia and the Pacific did not meet the recommended two servings of fruit per day and about 48 % of girls consumed less than three servings of vegetables per day^(38)^. Adolescent girls living in urban and peri-urban areas consumed more energy per day than girls from urban slums or rural areas. This pattern may indicate an epidemiologic pattern of nutrition transition that is affecting nations across the globe^(38)^. Unfortunately, our limited sample impedes observations on age and residential differences. However, our findings on energy and fruit and vegetable inadequacy suggest changes in adolescent eating behaviours and/or food patterns contribute to the increasing double burden of malnutrition in Indonesia^(9)^.

Previous evidence in Indonesia indicates shifting consumption towards energy-dense foods and/or beverages among Indonesians^(39)^, while dietary guidelines are generally unmet^(40)^. We found more than half of children and adolescents 6–18 years consumed more Na than the daily recommended amount, and 50 % of the consumption was sourced from food^(31)^. Concern for high Na intake stems from its association with CVD^(41)^ and its contribution to death from CVD^(42)^. Although we do not find local evidence of association between high Na intake and mortality, studies in Indonesia have found an association between high Na intake and elevated blood pressure, a recognised risk factor of CVD^(43,44)^. A prospective cohort study in Bogor, Indonesia found that adults who consumed more than or equal to 2 g/d of Na had higher incidences of hypertension compared with those consuming < 2 g/d^(44)^. The combination of diets high in Na and low in K – which is highest in fruits and vegetables – puts adolescents at particularly high risk of high-blood pressure and associated CVD later in life^(45)^. Further, a study in our review reported that sugary beverages were the second most commonly consumed beverages after water among 10–17 year olds, and females recorded higher consumption than males^(30)^. This finding is aligned with evidence in other LMIC where energy-dense foods, including sweet and salty items, are widely consumed. Half of adolescent girls (51 %) in LMIC consume sugar-sweetened beverages. Of girls who consumed them, half (49 %) consumed them 4–6 times/week and 46 % consumed them 2–3 times/week^(38)^. Our findings suggest adolescent diets in Indonesia need to be shifted from energy-dense, nutrient-poor processed foods to nutrient-rich, minimally processed foods. More research is needed to determine how to accomplish this, especially since geographical and cultural aspects are highly variable in Indonesia, and different approaches may be necessary depending on the local context.

Additionally, our review found that skipping breakfast^(22,33–36)^ and snacking were common among Indonesian adolescents^(36,37)^, which is typical for adolescents in LMIC^(38)^. Included studies observed consistent patterns that overweight/obese adolescents tend to skip breakfast and snack more than their normal-weight counterparts^(33,36)^. This is similar to findings from a meta-analysis which reported a positive association of skipping breakfast with the prevalence of overweight or obesity in Asia and Pacific regions^(46)^. Snacking is also associated with skipping breakfast among adolescents: females and adolescents living in metropolitan areas skipped breakfast more than those living in rural areas^(47)^. While studies showing association do not demonstrate that skipping breakfast or snacking causes overeating or overweight and obesity, there is a plausible mechanism that may explain a causal relationship. Adolescents that skip breakfast may be more likely to consume unhealthy foods that facilitate overeating when snacking, due to the context in which they snack. For example, they may have junk food or processed foods at school instead of a home-cooked meal or fresh fruits and vegetables. The impact of snacking on overweight and obesity may have to do primarily with the quality of the snack. A snack containing an egg and fresh fruits and vegetables contains a much higher nutrient density, fibre and protein content as well as lower energy density than most common snack foods and is unlikely to facilitate overeating. Foods available in adolescent environments, such as schools, are a gap that needs to be explored. More research is needed to understand how to implement effective dietary interventions targeted to adolescents that reduce all forms of malnutrition, including undernutrition, overweight/obesity and related noncommunicable diseases.

Our findings highlight the poor diet quality of Indonesian adolescents, reflecting an urgent need for effective programmes and interventions with adequate monitoring and evaluation. School-based programmes are easily implemented and generally reach a broad group of adolescents, yet out-of-school adolescents remain a particularly vulnerable group^(18)^. Promoting healthy nutrition and obesity prevention is necessary. Promotion measures have been shown to improve knowledge and boost positive attitudes towards healthy diets; however, its effectiveness on behaviour change is mixed^(48)^. Globally, there is growing interest in mobile-based interventions, especially social media, for its potential for a greater reach with less cost; however, to our knowledge there is no strong evidence for its effectiveness yet^(49)^. Thus, the type of intervention that fits the Indonesian adolescent profile needs to be explored through qualitative research, with particular attention on youth involvement and empowerment. On a broader view, a life cycle approach may be an ideal approach, which requires a supportive health system^(50)^ and conducive collaboration between policy makers, health service providers and development partners^(18,50)^.

A recent systematic review of the determinants of undernutrition among adolescents in LMIC showed that undernutrition is associated with determinants at the personal level (age, sex, birth order, religion, ethnicity, educational and literacy level, working status and marital status), household level (parental education and occupation, household size and composition, income and socio-economic status) and community level (residence, sanitation, school type and seasonality)^(51)^. In Indonesia, the challenge is heightened by different social and environmental conditions. Per our findings, all levels of governments in Indonesia should find a way to make everyone realise the critical role that nutrition plays in adolescents’ life. It is crucial to provide a thorough information about the urgency of nutrition and health problems of adolescents in Indonesia and why it needs to be intervened immediately. This dissemination of information should be conducted to every layer of the community, not only to adolescents, to be able to provide a supportive environment for adolescents to change their behaviour towards a healthier one. Our review found that many adolescents are skipping breakfast, and unhealthy snacking is still a widespread practice in Indonesian adolescents. We need to change not only the knowledge of these adolescents but also their attitude and behaviour towards a healthier diet. Again, any intervention programmes should aim at adolescents and their social environments. For example, when an adolescent is keen to change their eating pattern to a healthier diet, this cannot be achieved when the parents are not equipped with similar knowledge and ability to provide healthier choices at home. Within this situation, the school also plays a critical role in providing healthier meals and snacks as well as beverages in the school environment.

The next step is building a programme to disseminate information towards Indonesian adolescents and their supportive environments about adolescents’ health in general and nutrition education in specific. Nutrition education should include specific knowledge on diet quality and fruit and vegetable consumption, as already highlighted in the ‘*Isi Piringku*’ initiatives stated in the Ministry of Health Law no. 41 in 2014^(52)^. Considering that the rate of in-school adolescents in Indonesia is high, the school channel is still the most strategic way to intervene. This intervention programme should target adolescents in all areas in Indonesia since living in urban areas did not guarantee that adolescents will have better nutrition knowledge. Our findings highlight that adolescents in urban and peri-urban areas are found to be consuming more energy daily compared with their rural counterparts. Another essential step for the government to take is to consider a way to educate adolescents through a role model for a ‘healthier lifestyle’. This role model should be able to convey messages to reduce the consumption of salt and sugar-sweetened beverages, as well as how to support adequate nutrition with physical activity.

Importantly, all currently implemented strategies need to be closely monitored and evaluated for their generalisability, given Indonesia’s highly unique and diverse context. Several surveys in Indonesia already collect some dietary indicators, for example, fruit and vegetable consumption and household food consumption from national surveys like the basic health research and national socio-economic surveys. However, we need to check whether these indicators are in line with the global updates and standards, so they will be comparable to national surveys of other countries. For instance, one could use the global individual food consumption data tool by FAO – WHO as a reference^(53)^. These tools tailor questions for different needs, including health, nutrition and agricultural policy. Adopting such global reference would address the concern on assessment quality issues while ensuring findings are comparable to other countries.

To our knowledge, ours is the first review that gathers extensive evidence of adolescent eating patterns in Indonesia. A strength of the current study is that we performed a systematic literature search of studies in both English and Indonesian languages. The use of a tool to critically appraised studies ensured that only high-quality studies were included in this review. This tool also reduced subjectivity and bias. Lastly, despite the comprehensive search, our findings and conclusion need to be interpreted with acknowledgment of some limitations. First, direct quantitative comparisons between similar studies are not feasible because the different definitions and or measurements of eating pattern variables used by each study. This difference also limited comparisons between age groups where different eating patterns among younger and older adolescents were reported^(38)^. This limitation is also reported by other systematic reviews^(38,54,55)^, with similar reasons, including the paucity of data as well as the confirmed definition or variables in studies among Indonesian adolescents. Second, limited nationwide information limits the ability to generalise findings across Indonesia and to compare subgroups adequately. Third, we acknowledge that since some of the surveys we included in our review were conducted more than 10 years ago, dietary patterns may have changed in the last decade. Lastly, although we realise that food patterns or intake cannot be dissociated from nutritional status, only three out of the included studies depicted nutritional status. Therefore, we were unable to analyse the association between nutritional status and food intake.

This review also highlights the importance of better definitions and measurement tools for eating patterns in Indonesia, especially for adolescents. Standardised definitions and tools for dietary patterns are essential for all levels of government so that results from the available country-level surveys currently in place as well as future surveys can more easily be compared and utilised.

The government should endorse future research that explores the possible differences between sex, age groups (younger, older adolescents) and residents (urban, rural and urban slum). Future studies should also aim to gather evidence using cohort methods, allowing us to monitor trends and changes in eating behaviour among adolescents. Furthermore, a deep understanding of facilitators, barriers and motives of adolescents’ food choices will be valuable to inform policymakers on effective interventions. Sustained and evidence-based measures are needed to adequately inform public health policies and programmes in this fourth most populated nation.

## Supplementary material

For supplementary material accompanying this paper visit https://doi.org/10.1017/S1368980020002876. click here to view supplementary material
