# Supplementary material for: Eating behaviour of Indonesian adolescents: a systematic review of the literature
Source: Public Health Nutr. 2020 Sep 10;24(Suppl 2):s84–97. doi: 10.1017/S1368980020002876 (PMC10071220; doi:10.1017/S1368980020002876)
Supplement: Supplementary file 1 [file S1368980020002876sup001.docx]

Supplementary Table 1. Guidelines for critically appraising studies of prevalence or incidence of a health problem by Loney at al. ^(21)^

| Criteria | Scoring system |
| --- | --- |
| A. ARE THE STUDY METHODS VALID? |  |
| - Are the study designs and sampling method appropriate for the research question? | 1 point |
| - Is the sampling frame appropriate? | 1 point |
| - Is the sample size adequate? | 1 point |
| - Are objectives, suitable and standard criteria used for measurement of the health outcome? | 1 point |
| - Is the health outcome measured in an unbiased fashion? | 1 point |
| 6. Is the response rate adequate? Are the refusers described? | 1 point |
| B. WHAT IS THE INTERPRETATION OF THE RESULTS? |  |
| 7. Are the estimates of prevalence or incidence given with confidence intervals and in detail by subgroup, if appropriate? | 1 point |
| C. WHAT IS THE APPLICABILITY OF THE RESULTS? |  |
| 8. Are the study subjects and the setting described in detail and similar to those of interest to you? | 1 point |
| Total | 8 points |
